# Prognostic significance of the combination of preoperative red cell distribution width and platelet distribution width in patients with gastric cancer

**DOI:** 10.1186/s12885-021-09043-5

**Published:** 2021-12-09

**Authors:** Hiroaki Saito, Shota Shimizu, Yuji Shishido, Kozo Miyatani, Tomoyuki Matsunaga, Yoshiyuki Fujiwara

**Affiliations:** 1 Department of Surgery, Japanese Red Cross Tottori Hospital, 117 Shotoku-cho, Tottori, 680-8517 Japan; 2 Division of Gastrointestinal and Pediatric Surgery, Department of Surgery, School of Medicine, Tottori University Faculty of Medicine, 36-1 Nishi-cho, Yonago, 683-8504 Japan

**Keywords:** Gastric cancer, Platelet distribution width, Prognosis, Recurrence, Red cell distribution width

## Abstract

**Background:**

Platelet distribution width (PDW) and red cell distribution width (RDW) are readily obtainable data, and are reportedly useful as prognostic indicators in some cancers. However, their prognostic significance is unclear in gastric cancer (GC).

**Methods:**

We enrolled 445 patients with histopathological diagnoses of gastric adenocarcinoma who had undergone curative surgeries.

**Results:**

According to the optimal cut-off value of PDW and RDW by receiver operating characteristic (ROC) analysis, we divided patients into PDW^High^ (≥ 16.75%), PDW^Low^ (< 16.75%), RDW^High^ (≥ 14.25%), and RDW^Low^ (< 14.25%) subgroups. Overall survival (OS) was significantly worse in patients with PDW^High^ than in those with PDW^Low^ (*P* = 0.0015), as was disease specific survival (*P* = 0.043). OS was also significantly worse in patients with RDW^High^ than in those with RDW^Low^ (*P* <  0.0001), as was disease specific survival (*P* = 0.0002). Multivariate analysis for OS revealed that both PDW and RDW were independent prognostic indicators. Patients were then given PDW-RDW score by adding points for their different subgroups (1 point each for PDW^High^ and RDW^High^; 0 points for PDW^Low^ and RDW^Low^). OS significantly differed by PDW-RDW score (*P* <  0.0001), as did disease specific survival (*P* = 0.0005). In multivariate analysis for OS, PDW-RDW score was found to be an independent prognostic indicator.

**Conclusions:**

The prognosis of GC patients can be precisely predictable by using both PDW and RDW.

## Background

Recently, there has been growing interest in developing prognostic indicators for various cancers using hematological and serologic parameters, which can be obtained easily and less invasively. Since complete blood count (CBC) data is easily obtainable data in routine clinical setting, many prognostic indicators, including neutrophil to lymphocyte ratio and platelet to lymphocyte ratio, have been developed using CBC data thus far.

Platelet distribution width (PDW) and red cell distribution width (RDW) are also data that can be obtained from CBC. PDW shows variation of platelet size distribution and is used to evaluate platelet morphology and activation [1, 2]. A recent study revealed close correlation among PDW, white blood cell count, and serum C-reactive protein level, indicating that PDW also reflect inflammation status [3]. The RDW shows the heterogeneity in erythrocyte size and is widely used as parameter for anemia [4]. Chronic inflammation and poor nutrition are thought to increase RDW, indicating that RDW also reflect inflammation and nutritional status [5–7]. Because both inflammation and nutritional status are reportedly associated with cancer prognosis, PDW and RDW are being recently studied as prognostic indicators for cancer patients.

Gastric cancer (GC) is the fifth leading cause of cancer deaths worldwide [8]. Although there are a few reports showing the prognostic significance of either PDW or RDW alone in GC patients, the prognostic significance of the combination of PDW and RDW has not been reported thus far. Because both PDW and RDW are obtainable from preoperative CBC data, the combination of PDW and RDW might provide useful prognostic information in managing GC patients including treatment strategy preoperatively. Here we conducted this study to answer this question.

## Methods

### Patients

A total of 445 gastric cancer patients who underwent gastrectomy at Tottori University Hospital between January 2005 and December 2013 were included in this study. The inclusion criteria were as follows: patients (1) with newly histologically confirmed gastric adenocarcinoma; and (2) underwent curative gastrectomy (R0 resection). The exclusion criteria were as follows: patients (1) had synchronous or metachronous cancer in other organs; (2) underwent neoadjuvant chemotherapy; and (3) without complete medical records and available follow-up data. The Japanese Classification of Gastric Carcinoma was used to determine their clinicopathologic findings [9]. The number of patients were 310, 82, and 53 in stage I, II, and IIII, respectively. Patients periodically visited outpatient clinics to take blood test including tumor marker and diagnostic imaging, such as esophagogastroduodenoscopy, ultrasonography, and computed tomography, for early detection of recurrence. PDW, RDW, serum level of carcinoembryonic antigen (CEA), and platelet count (PC) in the peripheral blood, which were measured within 1 month before operation, patterns of recurrence, and causes of death were obtained through review of the hospital database. For the measurement of PDW and RDW, blood samples were collected in tubes containing dipotassium ethylenediaminetetraacetic acid as an anti-coagulant and analyzed immediately after collection.

### Statistical analysis

The chi-squared test was used to determine the differences in clinicopathologic characteristics between groups. The optimal cutoffs for preoperative RDW and PDW in the survival analysis were determined using the receiver operating characteristic (ROC) analysis. In the survival analysis, OS refers to the time which begins at operation and up to the time of death. All causes of death are included to calculate OS. The disease specific survival (DSS) was defined as the interval between surgery and the date of death from gastric cancer. Therefore, the deaths not caused by gastric cancer were considered as lost to follow-up as of time of death for the statistical analysis of disease specific survival rate. Survival curves were constructed using Kaplan–Meier method. Their differences were determined using the log-rank test. Multivariate analysis was performed using Cox’s proportional hazards model and a stepwise procedure. The covariates included in this study are age, gender, tumor size, histology, depth of invasion, lymph node metastasis, lymphatic invasion, vascular invasion, approach (open or laparoscopy), type of gastrectomy, lymph node dissection, adjuvant chemotherapy, PDW, and RDW. *P* <  0.05 was considered statistically significant. GraphPad Prism (GraphPad Software, Inc., La Jolla, CA, USA) and Stat View (Abacus Concepts, Inc., Berkeley, CA, USA) software were used for the statistical analyses.

## Results

**Fig. 1:**
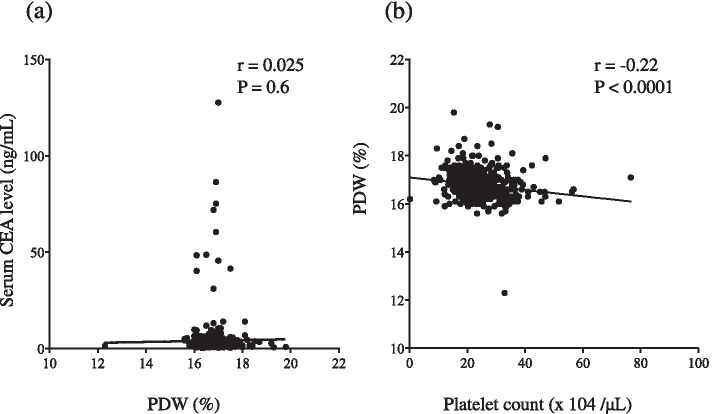
**a** Platelet distribution width (PDW) and serum CEA level were not significantly correlated (*r* = 0.025, *P* = 0.6); however (**b**), PDW and peripheral platelet count (PC) were significantly but weakly correlated (*r* = ^−^ 0.22, *P* <  0.0001)

**Fig. 2:**
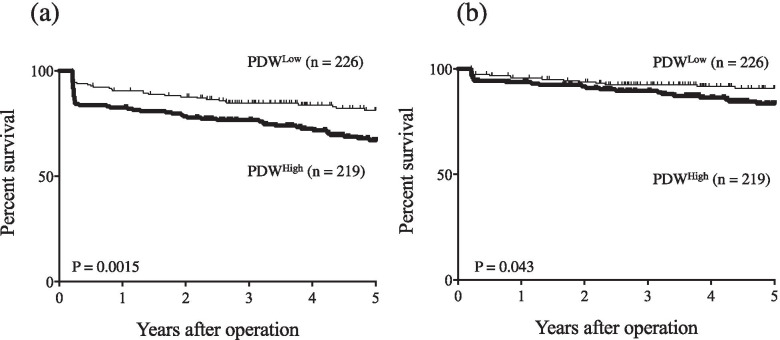
**a** Overall survival (OS) curves by preoperative PDW. OS was significantly worse in PDW^High^ patients than in PDW^Low^ patients (*P* = 0.0015). **b** Disease-specific survival (DSS) curves by PDW. DSS was significantly worse in PDW^High^ patients than in PDW^Low^ patients (*P* = 0.043)

The mean PDW was 16.78% (range: 12.30–19.8%). Figure 1 shows the correlation among PDW, CEA and PC. PDW and CEA were not significantly correlated (*r* = 0.025; *P* = 0.6); however, PDW and PC were significantly but weakly correlated (*r* = ^−^ 0.22; *P* <  0.0001). ROC curve for OS status revealed the area under the curve (AUC) of PDW was higher than that of PC, which indicates that PDW is a better prognostic indicator than PC for GC patients. Because ROC analysis for OS indicated that the optimal cut-off value of PDW was 16.75% (AUC = 0.583, *P* = 0.011), we divided patients into PDW^High^ (≥ 16.75; *n* = 219) and PDW^Low^ (< 16.75; *n* = 226). Table 1 shows the correlation between preoperative PDW and patients’ clinicopathological variables. Preoperative PDW^High^ was significantly more common in elderly patients (≥ 70 years) than in non-elderly patients (< 70 years; *P* = 0.0014). OS was significantly worse in patients with PDW^High^ than in those with PDW^Low^ (*P* = 0.0015, Fig. 2a), as was DSS (*P* = 0.043, Fig. 2b).

**Table 1:** Comparison of patient characteristics versus preoperative platelet distribution width (PDW)

Variables	PDW^High^ (*n* = 219)	PDW^Low^ (*n* = 226)	*P* value
Age (years)			0.0014
< 70 (*n* = 217)	90 (41.5%)	127 (58.5%)	
≥ 70 (*n* = 228)	129 (56.6%)	99 (43.4%)	
Gender			0.23
Male (*n* = 328)	167 (50.9%)	161 (49.1%)	
Femal (*n* = 117)	52 (44.4%)	65 (55.6%)	
Tumor size (cm)			0.52
< 4 (*n* = 281)	135 (48.0%)	146 (52.0%)	
≥ 4 (*n* = 164)	84 (51.2%)	80 (48.8%)	
Histology ^a^			0.059
Differentiated (*n* = 244)	130 (53.3%)	114 (46.7%)	
Undifferentiated (*n* = 201)	89 (44.3%)	112 (55.7%)	
Depth of invasion ^b^			0.96
T1 (*n* = 284)	140 (49.3%)	144 (50.7%)	
T2/3/4 (*n* = 161)	79 (49.1%)	82 (50.9%)	
Lymph node metastasis			0.6
Absent (*n* = 342)	166 (48.5%)	176 (51.5%)	
Present (*n* = 103)	53 (51.5%)	50 (48.5%)	
Lymphatic invasion			0.5
Absent (*n* = 186)	88 (47.3%)	98 (52.7%)	
Present (*n* = 259)	131 (50.6%)	128 (49.4%)	
Vascular invasion			0.88
Absent (*n* = 232)	115 (49.6%)	117 (50.4%)	
Present (*n* = 213)	104 (48.8%)	109 (51.2%)	
Stage of disease			0.77
I (*n* = 310)	154 (49.7%)	156 (50.3%)	
II / III (*n* = 135)	65 (48.1%)	70 (51.9%)	
Approach			0.77
Open (*n* = 257)	129 (50.2%)	128 (49.8%)	
Laparoscopy (*n* = 188)	90 (47.9%)	98 (52.1%)	
Gatrectomy (total vs. distal and proximal partial)		0.073	
Total (*n* = 95)	39 (41.1%)	56 (58.9%)	
Distal or proximal (*n* = 350)	180 (51.4%)	170 (48.6%)	
Lymph node dissection			0.76
D0 / D1 (*n* = 312)	155 (49.7%)	157 (50.3%)	
D2 (*n* = 133)	64 (48.1%)	69 (51.9%)	
Adjuvant chemotherpy			0.36
Absent (*n* = 377)	189 (50.1%)	188 (49.9%)	
Present (*n* = 68)	30 (44.1%)	38 (55.9%)	

^a^Differentiated or undifferentiated; Differentiated, papillary or tubular adenocarcinoma; undifferentiated, poorly differentiated or mucinous adenocarcinoma, or signet-ring cell carcinoma

^b^Depth of invasion: T1, tumor invasion of the lamina propria or submucosa; T2, tumor invasion of the muscularis propria; T3, tumor invasion of the subserosa; T4, tumor penetration of the serosa or tumor invasion of adjacent organs

**Fig. 3:**
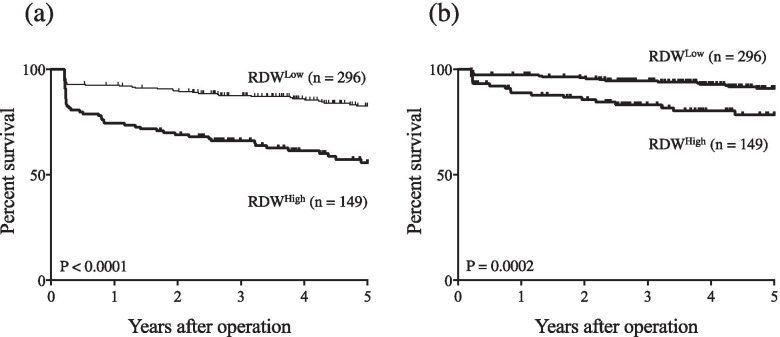
**a** Overall survival (OS) curves by preoperative RDW. OS was significantly worse in RDW^High^ patients than in RDW^Low^ patients (*P* <  0.0001). **b** Disease-specific survival (DSS) curves by preoperative RDW. DSS was significantly worse in RDW^High^ patients than in RDW^Low^ patients (*P* = 0.0002)

Because ROC analysis for OS indicated that the optimal cut-off value of RDW was 14.25% (AUC = 0.651, *P* <  0.0001), we divided patients into RDW^High^ (≥ 14.25; *n* = 149) and RDW^Low^ (< 14.25%; *n* = 296). Table 2 shows the correlation between preoperative RDW and patients’ clinicopathological variables. Preoperative RDW^High^ was significantly more common in elderly patients, those with larger tumors, and those with vascular invasion than in non-elderly patients (*P* = 0.0032), those with smaller tumors (*P* = 0.0004), and those without vascular invasion (*P* = 0.011), respectively. OS was significantly worse in patients with RDW^High^ than in those with RDW^Low^ (*P* <  0.0001, Fig. 3a), as was DSS (*P* = 0.0002, Fig. 3b). Univariate analysis revealed that age, tumor size, depth of invasion, lymph node metastasis, lymphatic invasion, vascular invasion, approach, type of gastrectomy, lymph node dissection, adjuvant chemotherapy, preoperative PDW and RDW were significantly associated with OS. In multivariate analysis for OS, both PDW and RDW were independent prognostic indicators in GC patients, along with age, approach, lymph node metastasis, and vascular invasion (Table 3).

**Table 2:** Comparison of patient characteristics versus preoperative red cell distribution width (RDW)

Variables	RDW^High^ (*n* = 149)	RDW^Low^ (*n* = 296)	*P* value
Age (years)			0.0032
< 70 (*n* = 217)	58 (26.7%)	159 (73.3%)	
≥ 70 (*n* = 228)	91 (39.9%)	137 (60.1%)	
Gender			0.47
Male (*n* = 328)	113 (34.5%)	215 (65.5%)	
Femal (*n* = 117)	36 (30.8%)	81 (69.2%)	
Tumor size (cm)			0.0004
< 4 (*n* = 281)	77 (27.4%)	204 (72.6%)	
≥ 4 (*n* = 164)	72 (43.9%)	92 (56.1%)	
Histology			0.39
Differentiated (*n* = 244)	86 (35.2%)	158 (64.8%)	
Undifferentiated (*n* = 201)	63 (31.3%)	138 (68.7%)	
Depth of invasion ^a^			0.057
T1 (*n* = 284)	86 (30.3%)	198 (69.7%)	
T2/3/4 (*n* = 161)	63 (39.1%)	98 (60.9%)	
Lymph node metastasis			0.55
Absent (*n* = 342)	112 (32.7%)	230 (67.3%)	
Present (*n* = 103)	37 (35.9%)	66 (64.1%)	
Lymphatic invasion			0.059
Absent (*n* = 186)	53 (28.5%)	133 (71.5%)	
Present (*n* = 259)	96 (37.1%)	163 (62.9%)	
Vascular invasion			0.011
Absent (*n* = 232)	65 (28.0%)	167 (72.0%)	
Present (*n* = 213)	84 (39.4%)	129 (60.6%)	
Stage of disease			0.54
I (*n* = 310)	101 (32.6%)	209 (67.4%)	
II / III (*n* = 135)	48 (35.6%)	87 (64.4%)	
Approach			0.16
Open (*n* = 257)	93 (36.2%)	164 (63.8%)	
Laparoscopy (*n* = 188)	56 (29.8%)	132 (70.2%)	
Gatrectomy (total vs. distal and proximal partial)			0.13
Total (*n* = 95)	38 (40.0%)	57 (60.0%)	
Distal or proximal (*n* = 350)	111 (31.7%)	239 (68.3%)	
Lymph node dissection			0.92
D0 / D1 (*n* = 312)	104 (33.3%)	208 (66.7%)	
D2 (*n* = 133)	45 (33.8%)	88 (66.2%)	
Adjuvant chemotherpy			0.14
Absent (*n* = 377)	121 (32.1%)	256 (67.9%)	
Present (*n* = 68)	28 (41.2%)	40 (58.8%)	

See Table 1 for histology and the depth of invasion

**Table 3:** Univariate and multivariate analysis for overall survival of gastric cancer patients using Cox proportional hazard model and a stepwise procedure

	Univariate			Multivariate		
	*P* value	Hazard ratio	95% CI	*P* value	Hazard ratio	95% CI
Age (≥ 70 vs. < 70)	< 0.0001	4.968	3.206–7.700	< 0.0001	3.915	2.515–6.093
Gender (male vs. female)	0.1855	1.392	0.853–2.273			
Tumor size (≥ 4 cm vs. < 4 cm)	< 0.0001	2.604	1.751–3.861			
Histology (undifferentiated vs. differentiated)	0.4867	1.105	0.776–1.703			
Depth of invasion (T2/T3/T4 vs. T1)	< 0.0001	2.599	1.750–3.858			
Lymph node metastasis (present vs. absent)	< 0.0001	3.096	2.090–4.584	0.0042	1.923	1.229–3.010
Lymphatic invasion (present vs. absent)	< 0.0001	2.487	1.573–3.934			
Vascular invasion (present vs. absent)	< 0.0001	3.332	2.163–5.134	0.0114	1.898	1.155–3.119
Approach (open vs. laparoscopy)	< 0.0001	3.140	1.857–5.309	0.0238	1.888	1.088–3.275
Gatrectomy (total vs. distal and proximal partial)	0.0002	2.237	1.471–3.401			
Lymph node dissection (D2 vs. D0/D1)	0.0166	1.623	1.092–2.410			
Adjuvant chamotherapy (present vs. absent)	0.0034	1.939	1.245–3.018			
Preoperative PDW (PDW^High^ vs. PDW^Low^)	0.0020	1.900	1.266–2.853	0.0009	2.031	1.338–3.082
Preoperative RDW (RDW^High^ vs. RDW^Low^)	< 0.0001	2.882	1.946–4.268	< 0.0001	2.553	1.714–3.802

*CI* confidence interval

See Table 1 for histology and the depth of invasion

**Fig. 4:**
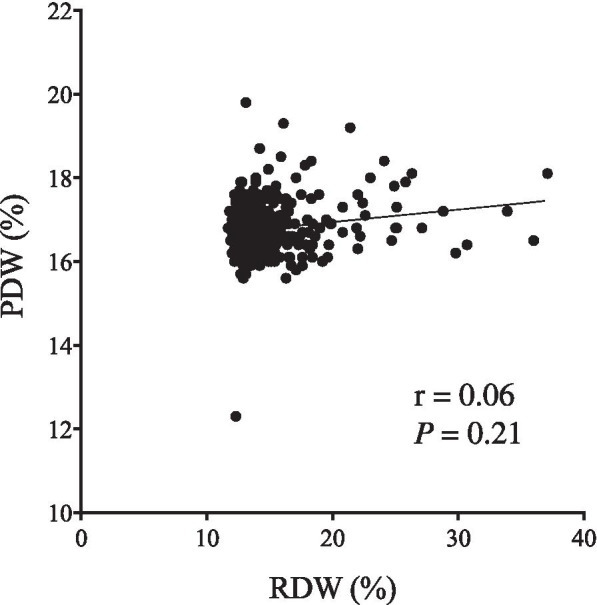
**a** The correlation between PDW and RDW (*r* = 0.06, *P* = 0.21)

**Fig. 5:**
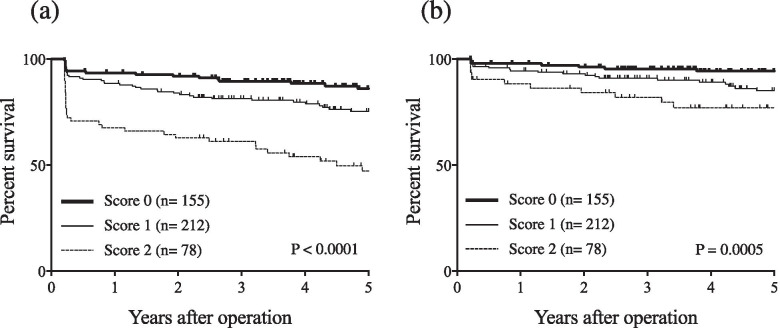
**a** Overall survival (OS) curves by PDW-RDW score. OS significantly differed by PDW-RDW score (*P* <  0.0001). **b** Disease specific survival (DSS) curves by PDW-RDW score. DSS significantly differed by PDW-RDW score (*P* = 0.0005)

As we saw no correlation between preoperative PDW and RDW (*r* = 0.06; *P* = 0.21; Fig. 4), we thought the combination of PDW and RDW might be more useful than either indicator used separately. Patients were then given PDW-RDW scores by adding points for their different subgroups (1 point each for PDW^High^ and RDW^High^; 0 points for PDW^Low^ and RDW^Low^). OS significantly differed by PDW-RDW score (*P* <  0.0001, Fig. 5a), as did DSS (*P* = 0.0005, Fig. 5b). In multivariate analysis for OS, PDW-RDW score was found to be an independent prognostic indicator (Table 4).

**Table 4:** Multivariate analysis for overall survival of gastric cancer patients using Cox proportional hazard model and a stepwise procedure

	*P* value	Hazard ratio	95% CI
Age (≥ 70 vs. < 70)	< 0.0001	3.922	2.519–6.107
Lymph node metastasis (present vs. absent)	0.0046	1.910	1.220–2.990
Vascular invasion (present vs. absent)	0.0079	1.948	1.191–3.187
Approach (open vs. laparoscopy)	0.0237	1.889	1.089–3.279
Combination of PDW and RDW	< 0.0001	2.292	1.697–3.096

*CI* confidence interval

## Discussion

Platelets have some pro-tumor effects and play significant roles in cancer progression and metastasis. They produce platelet derived endothelial cell growth factor (PD-ECGF) [10]. PD-ECGF was a well-described angiogenetic factor and reported to enhance neoangionenesis at tumor site, which promote tumor growth and metastases. Platelets also form aggregates with tumor cells in circulation, facilitating their adhesion to the vascular endothelium, which results in enhancement of metastasis, since tumor cell adhesion to the vascular endothelium is the important step for the formation of metastasis by tumor cells [11, 12]. Considering these pro-tumor effects of platelets, it is likely that platelet-related markers are useful prognostic indicators. In fact, the prognostic significance of PC has been reported in various cancers, including GC, thus far [13–17].

PDW is another platelet-related marker. Inflammation cytokines play essential roles in the development of inflammatory microenvironments in cancer, which contributes to the process of the tumor progression and metastasis. Excessive pro-inflammatory cytokines interfere with megakaryopoiesis, leading to an increased production of small-sized platelets from the bone marrow [18]. We previously demonstrated that the serum inflammation cytokine interleukin-6 (IL-6) level was significantly higher in the GC patients than in the healthy subjects [19]. Therefore, it is likely that PDW increases in GC patients under these situations. Although clinicians pay less attention to PDW than PC as a prognostic indicator, we found that the AUC of PDW for OS was higher than that of PC, indicating that PDW is a more accurate prognostic indicator than PC in GC patients. In fact, we demonstrated that elevated PDW was closely related to poor prognosis in GC patients in this study. The close correlation between elevated PDW and poor prognosis was also reported in patients with esophageal cancer [20], hepatocellular carcinoma [21], and breast cancer [22]. On the other hand, Cheng et al. demonstrated that decreased PDW was significantly associated with poor disease free survival for early GC, which was totally opposite to our results [23]. In their study, they used median value of PDW as cut-off. On the other hand, we determined optimal cut-off value by using ROC analysis. Furthermore, they determined early GC patients, while we determined both early and advanced GC. These might make differences between our results and their ones.

Recent studies showed the close correlation between high RDW and poor prognosis in patients with lung cancer [24]. The iron deficiency anemia is often observed in GC patients because GC is associated with chronic blood loss, poor nutrition, and low iron absorption. Cancer-induced inflammation leads to inhibited response to erythropoietin, reduced iron release from reticuloendothelial macrophages, and shortened red blood cell survival. The number of immature red blood cells in the periphery increases under these situations, which results in high RDW. Therefore, high RDW is often observed in GC patients. The close correlations among RDW, inflammation, and nutritional status let us speculate that RDW could be a prognostic indicator in cancer patients, as both inflammation and poor nutritional status are often observed in cancer patients and worsen their prognosis. In fact, Cheng et al. reported preoperative RDW as prognostic indicator in GC patients [23]. We also demonstrated that high RDW was significantly related to poor prognosis of GC patients. Furthermore, multivariate analysis revealed that both PDW and RDW were independent prognostic indicators in GC patients. Since there was no statistically significant correlation between preoperative RDW and PDW, we thought that the combination of PDW and RDW might be more useful than either indicator used alone. To confirm this possibility, we determined the prognostic significance of PDW-RDW score in this study and found that PDW-RDW score was more useful in predicting the prognosis of GC patients than single usage of either PDW or RDW alone. To our knowledge, this is the first study to demonstrate the prognostic significance of the combination of PDW and RDW in GC patients.

Our study had a few limitations. First, because it was retrospective, it was subject to bias. Second, the number of patients included in our study was small and the results must therefore be confirmed in a large-scale trial.

In conclusion, our study suggests the potential utility of combining PDW and RDW to predict prognosis in GC patients. Because they are obtainable from CBC data, the combination of PDW and RDW may be useful in managing GC patients in routine clinical settings.

## Data Availability

The datasets generated during and analysed during the current study are not publicly available due to their containing information that could compromise the privacy of research participants but are available from the corresponding author on reasonable request.

## Abbreviations

CEA: Carcinoembryonic antigen
CBC: Complete blood count
DSS: Disease-specific survival
GC: Gastric cancer
IL-6: Interleukin-6
OS: Overall survival
PC: Platelet count
PD-ECGF: Platelet derived endothelial cell growth factor
PDW: Platelet distribution width
RDW: Red cell distribution width
ROC: Receiver operating characteristic
